# CRISPRdigger: detecting CRISPRs with better direct repeat annotations

**DOI:** 10.1038/srep32942

**Published:** 2016-09-06

**Authors:** Ruiquan Ge, Guoqin Mai, Pu Wang, Manli Zhou, Youxi Luo, Yunpeng Cai, Fengfeng Zhou

**Affiliations:** 1 Shenzhen Institutes of Advanced Technology, and Key Lab for Health Informatics, Chinese Academy of Sciences, Shenzhen, Guangdong 518055, China; 2 Shenzhen College of Advanced Technology, University of Chinese Academy of Sciences, Shenzhen, Guangdong, 518055, China; 3 Center for Synthetic Biology Engineering Research, Shenzhen Institutes of Advanced Technology, Chinese Academy of Sciences, Shenzhen, Guangdong 518055, China; 4 School of Science, Hubei University of Technology, Wuhan, Hubei, 430068, China; 5 College of Computer Science and Technology, Changchun, Jilin, 130012, China; 6 Key Laboratory of Symbolic Computation and Knowledge Engineering of Ministry of Education, Jilin University, Changchun, Jilin 130012, China

## Abstract

Clustered regularly interspaced short palindromic repeats (CRISPRs) are important genetic elements in many bacterial and archaeal genomes, and play a key role in prokaryote immune systems’ fight against invasive foreign elements. The CRISPR system has also been engineered to facilitate target gene editing in eukaryotic genomes. Using the common features of mis-annotated CRISPRs in prokaryotic genomes, this study proposed an accurate *de novo* CRISPR annotation program CRISPRdigger, which can take a partially assembled genome as its input. A comprehensive comparison with the three existing programs demonstrated that CRISPRdigger can recover more Direct Repeats (DRs) for CRISPRs and achieve a higher accuracy for a query genome. The program was implemented by Perl and all the parameters had default values, so that a user could annotate CRISPRs in a query genome by supplying only a genome sequence in the FASTA format. All the supplementary data are available at http://www.healthinformaticslab.org/supp/.

Clustered regularly interspaced short palindromic repeats (CRISPRs) are essential genetic factors in prokaryotic genomes^1^, and actively acquire template sequences from invasive elements such as phages for sequence-specific cut later on^2^,^3^. These template foreign sequences vary in length from 24 to 48 bps, gapped by conserved repeats^4^,^5^. A CRISPR is usually transcribed by a neighbouring CRISPR-associated (Cas) gene binding to its leader region on the closely flanking region^5^. The CRISPR/Cas system serves as an anti-invasion immune mechanism in over 40% of sequenced prokaryotic genomes^6^.

The CRISPR/Cas system is attracting considerable attention as a eukaryotic genome-editing technology^7^,^8^,^9^ because it cuts specific sequence signals^10^. The most well-developed enzyme is the nuclease Cas9 from the bacterium *Streptococcus pyogenes*^11^, and it may lead the degenerative cut to any genomic locations with an appropriate guide RNA fragment^12^. Another widely-used genome editing technology, TALEN^13^, requires the researcher to synthesize a nuclease for each target genomic location, which is much more costly and time-consuming than the synthesis of only a guide RNA^14^. With the increasing target specificity requested from the clinical applications, a number of Cas9 mutants have been introduced with over 50-fold higher specificity^15^,^16^.

A few computer programs have been developed for the *de novo* detection of natural CRISPRs in prokaryotic genomes. After their discovery in the 1980 s^17^, CRISPRs have been detected in 47.14% of the 2,762 analysed prokaryotic genomes, with 1.47 CRISPRs per genome^6^. However, the prokaryotic genomes are sequenced at an accelerated rate, and 4,278 genomes were included as of 25 September 2015 in the NCBI Microbial Genome database^18^. Consequently the *de novo* annotation of CRISPRs in a newly completed prokaryotic genome is necessary for the better understanding of this immune system. PILER-CR^19^ was derived from the repeat detection program PILER^20^, and screens for CRISPRs in a small genome. CRT screens for exact k-mer/k-nucleotide repeats in a genome, and concatenates the neighbouring repeats into candidate CRISPRs^21^. CRISPRFinder uses Vmatch^22^ to detect consecutively localized repeats, and demonstrates better annotations of DR boundaries and short CRISPRs^4^.

This study proposes a new *de novo* CRISPR detection program, CRISPRdigger, and focused on detecting weak DR signals, which are usually missed by the current literature^23^. The *de novo* repeat detection program RepeatScout was utilized to find repeat copies within a range of sequence lengths. After these repeat copies were clustered into groups, weak DR copies were annotated with RepeatMasker by mapping the template DRs onto the genome. Comparison with the existing programs was based on the gold standard CRISPR annotation from the dbCRISPR database^6^, updated on 14 April 2014^6^. Our dbCRISPR includes all location and sequence information of DRs and spacers. It is more convenient and accurate comparison with other programs^24^,^25^. The experimental data suggest that CRISPRdigger may be a good complement to the existing tools. The following sections give descriptions of the data used in this study and the CRISPRdigger algorithm, followed by a baseline summary of the annotated CRISPRs in dbCRISPR, and a comprehensive comparison of CRISPRdigger with the existing programs.

## Material and Methods

### Data preparation

**Figure 1:**
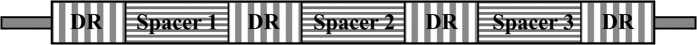
The schematic structure of a CRISPR. A number of spacer sequences (Spacers 1–3 in horizontal-line shaded boxes) are flanked by the highly conserved direct repeats (DRs in vertical-line shaded boxes).The DRs and spacers together constitute a CRISPR.

The curated dbCRISPR database was retrieved as the gold standard CRISPR annotation (denoted as GSD) on 14 April 2014^6^. The schematic structure of a CRISPR is demonstrated in Fig. 1, and its biological qualities have been comprehensively depicted in reviews^26^,^27^. There are 630 and 3,226 convincing CRISPRs which have been annotated for archaeal and bacterial genomes, respectively. The genera *Methanocaldococcus* and *Clostridium* have the largest CRISPR numbers for the kingdoms archaea and bacteria, respectively, and have been chosen as the representative microbial genomes to compare the proposed algorithm CRISPRdigger with the existing computer programs. The comparative results are summarized for the other microbial genus.

*Methanocaldococcus* and *Clostridium* have respectively 11 and 84 completely sequenced genomes in the NCBI database^28^. Eighty-five and 321 CRISPRs are annotated in six and 50 genomes, respectively, for the two genera in the dbCRISPR database updated on 14 April 2014^6^. The complete genomes of these microbes were downloaded from the NCBI database^28^ on 20 October 2014.

### CRISPRdigger screens for the DR signals of candidate CRISPRs

**Figure 2:**
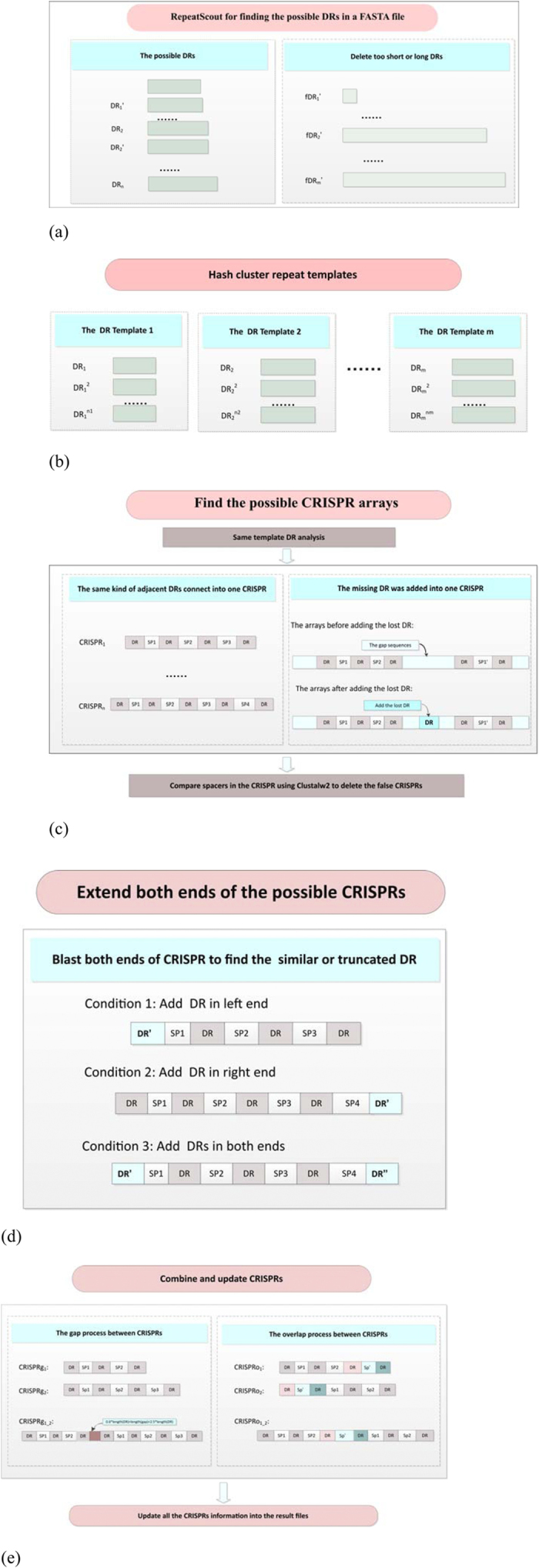
Five steps of the CRISPRdigger program. The direct repeats and spacers are abbreviated as DRs and SPs, respectively. (**a**) *De novo* screening for repeats within the known length distribution of DR signals. Only DRs within a short range are kept for the next step. (**b**) Clustering the template repeats into groups of candidate template DRs. The highly homologous direct repeats are regarded as one class. (**c**) Find the candidate CRISPR arrays. A series of consecutively distributed DR copies is combined as a candidate CRISPR, and two neighboring CRISPRs will be combined into one, if there are missing DR copies in between. Delete the false positives after comparing the similarity of the spacers. (**d**) Extend the two termini of the candidate CRISPRs. Flanking regions of annotated CRISPRs will also be screened for missing DR copies. (**e**) Combine and update the CRISPR annotations. Two cases of neighboring CRISPR pairs are processed. The first case is when the two neighboring CRISPRs are combined into one, if they share the same DR signal and the sequence length between them is within the spacer length distribution. The second case is when the two overlapping CRISPRs are combined into one. After these five steps, all the annotated CRISPRs were written into the result files.

The proposed algorithm CRISPRdigger uses RepeatScout for the *de novo* screening of repeats, and searches for the consecutively distributed repeat copies detected by RepeatMasker. The basic procedure is illustrated in Fig. 2, and the details may be found in the Supplementary Figure S1. CRISPRdigger accepts a FASTA file as its input.

The screening step of candidate DR signals uses a few refining techniques to detect the true positives, as shown in Fig. 2(a). Only the template repeats of lengths between 15 and 60 are kept for analysis, based on the length distribution of DR signals in the gold standard dbCRISPR database. Two low-complexity filters TRF^29^ and nseg^30^ are used with optimized parameters by RepeatScout to remove only the non-CRISPR low-complexity sequences. TRF is used with default parameters. Nseg is used with the parameter NSEG_THRESHOLD = 0.9, to make sure that CRISPRs which have DR signals of low sequence complexity are not removed.

The experimental data shows that RepeatScout may annotate the neighboring DR signals in the same CRISPRs as different repeats. Therefore the representative DR signals are selected as templates by using pair-wise dynamic programming to sort the highly similar DRs (similar ratio > 0.8) detected by RepeatScout into one kind of template DR, as shown in Fig. 2(b). RepeatMasker with the default parameters is used to map these new template DR signals onto the query genome.

Figure 2(c) shows that a list of candidate CRISPRs are generated from the consecutively distributed DR signals. It can be observed that two closely located CRISPRs have the same template DR and the sequence length between them is no more than the sum of the lengths of two spacers and one DR. Then all spacers in one CRISPR are aligned using ClustalW2^31^ with default parameters. The false positive CRISPRs are screened if they have highly similar spacers (similar ratio > 0.5). The terminal DR signals may also be missed by RepeatMasker for unknown reasons, as shown in Fig. 2(d), so an extra step with both the dynamic programming algorithm and BLAST is taken to screen for these missed DR signals.

All the above annotations are combined into the final list of CRISPR annotations, as shown in Fig. 2(e). The position annotations and sequences of the DR signals, spacers and complete CRISPRs are generated in tab-delimited text files. To facilitate the direct visualization of the annotated CRISPRs, the annotations are also provided in the format of GFF3.

### Prerequisite computer programs

The proposed algorithm is implemented as CRISPRdigger version 1.0 program, and the following programs are used to facilitate the CRISPR annotations. CRISPRdigger is implemented using the programming language Perl version 5.8.8 or later (http://www.perl.org/) and BioPerl version 1.6.901. The *de novo* repeat detecting program RepeatScout version 1.05^32^ is used to screen the candidate DR signals. The candidate DR signals are then mapped as templates to the investigated genome using the program RepeatMasker version 4.0.3^33^ with the database version 20140131. Our exploration of the candidate DR signals suggests that some DRs are very similar and may be grouped as copies of the same DR signal. The multiple sequence alignment program ClustalW2 version 2.1^31^ is used to detect false positive CRISPR candidates. The sequence alignment program NCBI BLAST+ version 2.2.28+^34^ is used to measure the similarity between two sequences.

For the convenience of users, a Perl script is provided for the automatic installation of all the prerequisite programs in the web site http://www.healthinformaticslab.org/supp/.

## Results and Discussion

### Baseline summary of dbCRISPR

CRISPRdigger tries to screen a query genome in the FASTA format for all the CRISPRs, based on the sequence parameters summarized from the gold standard dbCRISPR database.

**Figure 3:**
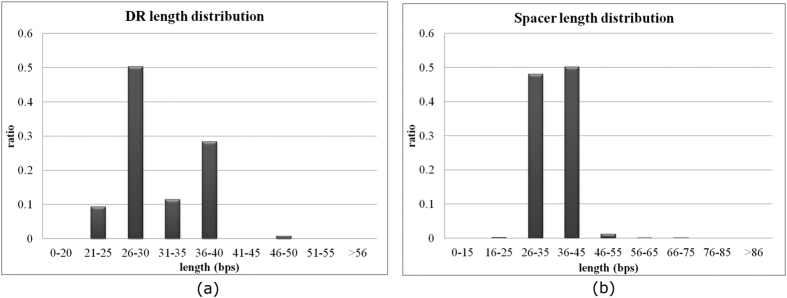
The histograms of the lengths of DRs and spacers in the dbCRISPR database. The lengths of (**a**) DRs and (**b**) spacers are measured in base pairs (bps).

The DR signals have an averaged length 31.32 bps within the range of 23 and 55 bps. Archaea CRISPRs tend to have shorter DR signals than bacteria. Both archaea and bacteria have DR signals as short as 23 bps, but the maximal DR length in archaea is 46 bps, compared with the maximal DR length 55 bps in bacteria. The averaged DR length in archaea is 29.32 bps, shorter than the averaged bacterial DR length 32.23 bps. The maximal archaeal DR length 46 is detected in *Candidatus Cloacamonas acidaminovorans* str Evry. This archaeal strain tends to have long DRs, and its shortest DR length 37 bps is longer than 73.02% of the archaeal genomes. The seven CRISPRs in the bacterium *Desulfobacca acetoxidans* DSM 11109 have the longest DR length of 55 bps, except for the fourth CRISPR with four 55-bp DRs, the other CRISPRs in *D. acetoxidans* DSM 11109 have an averaged DR length of only 37.45 bps. The histograms of the lengths of DRs and spacers of the CRISPRs in dbCRISPR are plotted in Fig. 3(a,b), respectively.

The database CRISPRdb has the same definition of DR lengths, *i.e.* 23–55 bps^6^. But another tool, CRISPRmap, employs a different range 19–48 bps for CRISPR DR lengths^25^. CRISPRmap considers that archaea has a shorter DR length range (20–44 bps) than bacteria (19–48 bps). Since the minimum DR length in the gold standard dbCRISPR database is 23 bps, this study didn’t utilize the lower bound 19 bps of CRISPRmap. CRISPRdigger utilizes a larger upper bound of DR lengths than CRISPRmap.

CRISPR spacers are usually captured from invasive genetic elements, and one CRISPR may acquire spacers from different sources, so unlike the DR signals, even spacers within the same CRISPR may have significantly different lengths. The prokaryotic CRISPR spacers have an average length of 35.64 bps, but the maximal length is as high as 857 bps in the dbCRISPR database. The longest spacer is observed in the bacterium *Clostridium novyi* NT. The firmicutes *C. novyi* NT harbours two CRISPRs with almost identical DR signals, and the longer CRISPR has 77 spacers, among which an 857-bp spacer is located. No other bacteria from the same genus have CRISPR spacers longer than 300 bps.

### Supporting evidence of our screening rules

**Figure 4:**
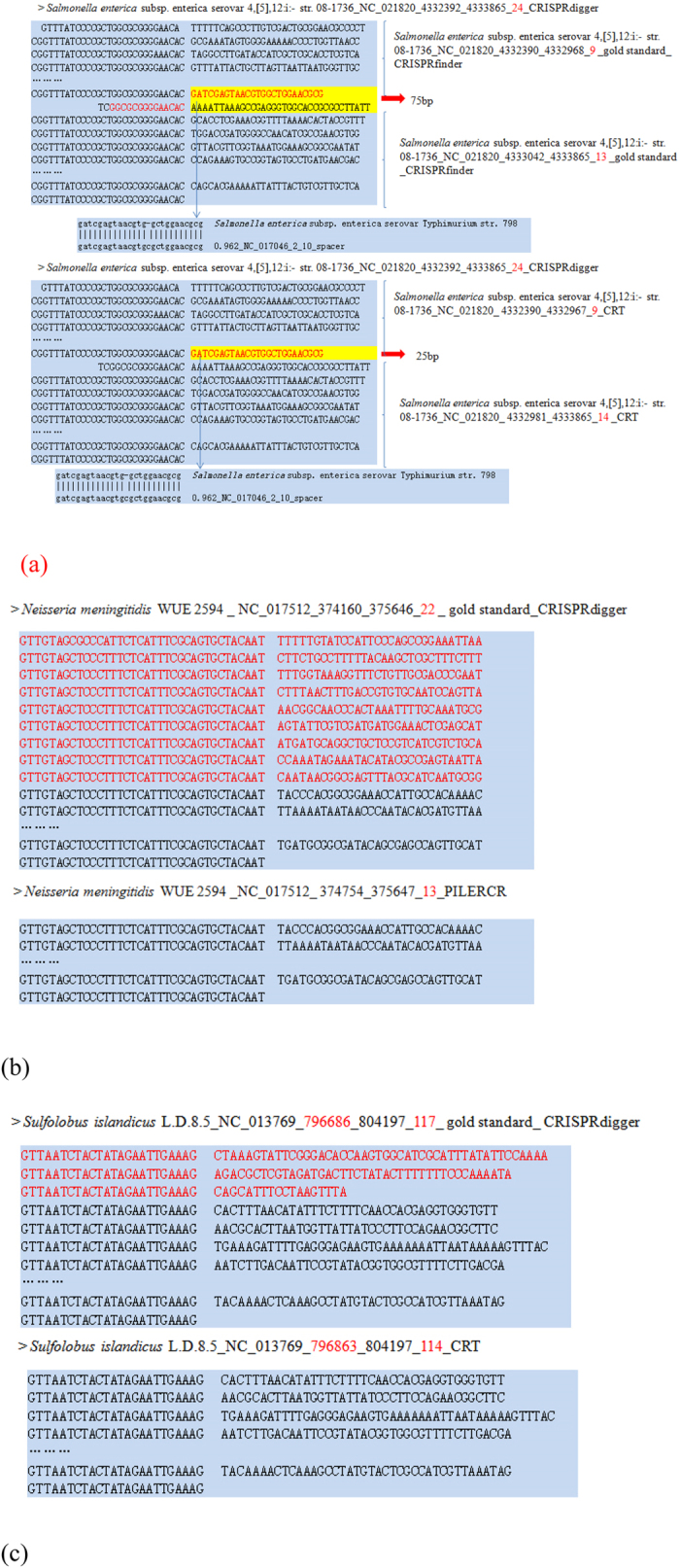
Mis-annotations of CRISPRs because of the missed DR signals. (**a**) The CRISPR with 24 spacers in *Salmonella enteric* subsp. Serovar 4,[5],12:i- str. 08–1736. This CRISPR was broken into two CRISPRs by CRISPRFinder and CRT in different ways. (**b**) Nine terminal DR signals in the 22-spacer CRISPR in *Neisseria meningitides* WUE 2594 were missed by the PILER-CR program. (**c**) Three terminal DR signals in a 117-spacer CRISPR in the archaea *Sulfolobus islandicus* L.D.8.5 were missed by CRT. The nucleotides missed by some CRISPR detection programs were highlighted in red.

Some DR signals may be missed by the repeat template mapping step of most programs. Figure 4(a) illustrates an example of a 24-spacer CRISPR in the pathogen *Salmonella enteric* subsp. Serovar 4,[5],12:i- str. 08–1736. However, CRISPRFinder did not find the partial DR signal in the CRISPR annotation, so that the 24-spacer CRISPR was annotated as two shorter CRISPRs with nine and thirteen spacers, respectively. The CRT program detected this partial DR signal, but did not concatenate the flanking 24 almost identical DR signals into one CRISPR. Therefore, the CRT program missed one spacer in this 24-spacer CRISPR. The terminal DR signals of a CRISPR may also be missed by some CRISPR annotation programs. CRISPRdigger correctly detected the gold standard 22-spacer CRISPR in *Neisseria meningitides* WUE 2594, but the PILER-CR program missed nine DR signals in the termini for unknown reasons, as shown in Fig. 4(b). These missed DR signals may be correctly recovered by PILER-CR using parameter “minid”≥0.98. So a user may want to test different parameter choices of CRISPR detection programs, if only a few target genomes are of interest.

Figure 4(c) gives one more example of terminal DR signals missed by a program. CRISPRdigger correctly detected the 118 DR signals of the CRISPR in *Sulfolobus islandicus* L.D.8.5, but CRT missed three terminal DR signals, probably because of a short spacer in between. This short spacer may be correctly recovered by CRT using different values for the parameter “minSL”, which further confirms the importance of parameter tuning for advanced computational users. Therefore, CRISPRdigger makes an extra effort to screen for candidate DR signals after the repeat template mapping procedure.

### Performance measurements

The following measurements are defined to evaluate how well a known CRISPR is recovered by a computer program. Because of its nature of repetitive structure, a simple measurement may not be sufficiently accurate to evaluate a CRISPR detection program. Some classification algorithms may be evaluated by sensitivity, specificity or area under the ROC curve^35^,^36^,^37^, but these performance measurements do not reflect the detection accuracy of the detailed CRISPR structures. Consequently a few novel performance measurements have been defined for this purpose. *NumDR* is defined as the number of DR copies detected by a given computer program in the location of a known CRISPR. For a given known CRISPR, the discovery ratio of DR copies *rDR* evaluates the ratio between the number of DR copies detected by a program and the DR copy number of the known CRISPR. *rDR* measures how complete a known CRISPR is detected by a program, and *rDR* may be greater than one if the program detects more DR copies than the dbCRISPR annotation. A summarized measurement *rCRISPR* is defined as the average *rDR* for all the known CRISPRs in a given genome.

**Figure 5:**
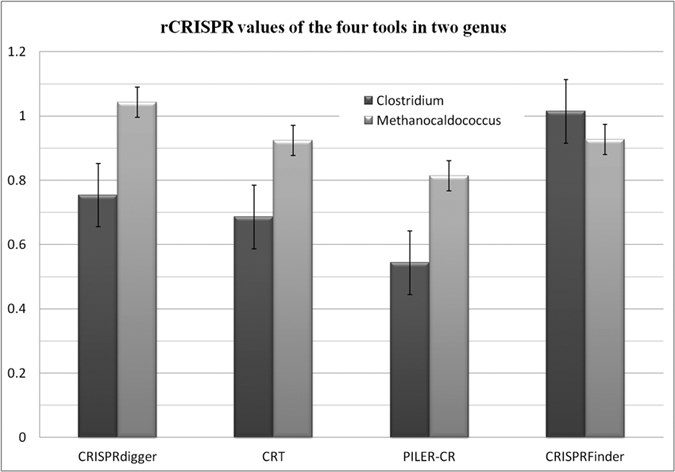
Comparison of the CRISPR prediction measurement rCRISPR by the CRISPRdigger, CRT, PILER-CR and CRISPRFinder programs. The ratio *rCRISPR* may be larger than 1.0, as more DR signals are predicted in the flanking regions of the CRISPR annotation from dbCRISPR.

CRISPRdigger is compared for CRISPR detection performance with the three existing CRISPR detection programs, *i.e.* CRT^21^, PILER-CR^19^ and CRISPRFinder^4^. The general measurement *rCRISPR* by the four computer programs for the top 10 genera with the largest CRISPR numbers in dbCRISPR are illustrated in the Supplementary Figure S2. The annotations of the top bacterial and archaeal genera are summarized in Fig. 5. In order to conduct a fair comparison, the DR lengths are set to 23–55 bps, and the spacer lengths are set to 10–120 bps. All the other parameters of CRT, PILER-CR and CRISPRFinder are set to their default values.

Based on the gold standard dbCRISPR annotation, CRISPRdigger achieves high values in the measurement *rCRISPR*, compared with the existing programs CRT, PILER-CR and CRISPRFinder. Figure 5 illustrates that the bacterium genus *Clostridium* has 50 genomes with at least one convincing CRISPR. The numbers of CRISPRs in the *Clostridium* genomes range between one and eighteen, and the DR numbers in the genomes are as large as 441. CRISPRdigger recovers 75.39% of the annotated DR signals on average, and outperforms CRT and PILER-CR by 6.81% and 21.02%, respectively. CRISPRFinder outperforms CRISPRdigger by 26.08% on the average *rCRISPR* measurement. CRISPRdigger performs the best on the archaeal genus *Methanocaldococcus*, and outperforms the other three programs CRT, PILER-CR and CRISPRFinder by 11.86%, 22.92% and 11.58%, respectively. Among the top 10 prokaryotic genera, CRISPRdigger performs the best for 5 genera and the second best for two other genera, as shown in the Supplementary Figure S2. Among the other three prokaryotic genus, CRISPRdigger achieves the average *rCRISPR* measurement 90.75% and 94.85%, slightly smaller than the best performances 93.00% (CRT on *Escherichia*) and 99.88% (CRISPRFinder on *Sulfolobus*), respectively. For the last genus *Streptococcus*, CRISPRdigger achieves 79.23% in *rCRISPR*, 20.81% lower than CRISPRFinder.

### Distribution of the DR discovery ratio *rDR*

**Figure 6:**
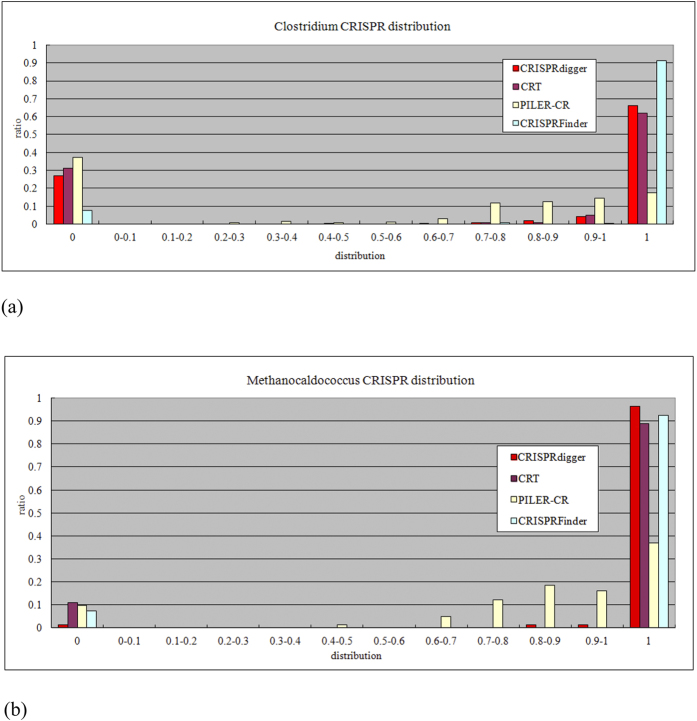
Distribution of the *rDR* measurement for all the CRISPRs. The histogram of (**a**) the bacterial genus *Clostridium*, and (**b**) the archaeal genus *Methanocaldococcus*.

The *rDR* measurement evaluates how complete a CRISPR is detected by a given computer program. Among the four CRISPR detection programs, CRISPRdigger ranks second best for the detection of CRISPRs in the bacterial genus *Clostridium*, as shown in Fig. 6(a). In the archaeal genus *Methanocaldococcus*, CRISPRdigger outperforms all the other three programs, as shown in Fig. 6(b). In both cases, the CRT and PILER-CR programs rank third and fourth, respectively. Supplementary Figure S2 illustrates that CRISPRdigger performs best or similarly compared with the best CRISPR detection programs regarding the top 10 prokaryotic genera with the largest CRISPR numbers.

### CRISPR annotations in two newly assembled genomes

**Figure 7:**
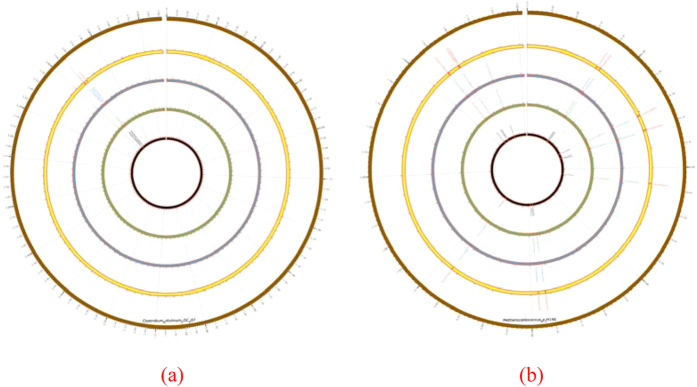
Circos plots of predicted CRISPRs in two recently completed microbial genomes. The plots are for (**a**) *Clostridium botulinum CDC*297 and (**b**) *Methanocaldococcus sp. JH*146, respectively. The outermost circle represents the chromosome of the microbe. The candidate CRISPRs detected by the PILER-CR, CRT, CRISPRFinder and CRISPRdigger programs are plotted on the four circles from innermost to the outermost, respectively. The text outside each circle gives the names of the annotated CRISPRs. The gap on the upper-side of each circle is the start and end positions of the chromosome.

Two recently completed genomes were selected for the comparative CRISPR annotations. By combining the candidate CRISPRs annotated by all the four programs, five CRISPRs could be detected in the *Clostridium botulinum CDC*297 bacterium and 11 in the archaea *Methanocaldococcus sp. JH*146. The CRISPR distributions were illustrated using the program Circos^38^, as shown in Fig. 7.

Similar results to those in the above sections were observed when the CRISPR annotations of the four programs were compared. PILER-CR missed three CRISPRs and split three long CRISPRs into shorter ones because of the undetected internal DRs. Four more CRISPRs were shortened by PILER-CR because of the undetected terminal DRs. CRT missed two CRISPRs. CRISPRFinder missed three CRISPRs. CRISPRdigger only missed the short CRISPR with four DRs between 1522152 and 1522397 bps of the chromosome of *Methanocaldococcus sp. JH*146. Generally, CRISPRdigger performs satisfactorily, and all the existing programs may co-operate to make a complete CRISPR annotation.

### Novel spacers detected by CRISPRdigger

**Figure 8:**
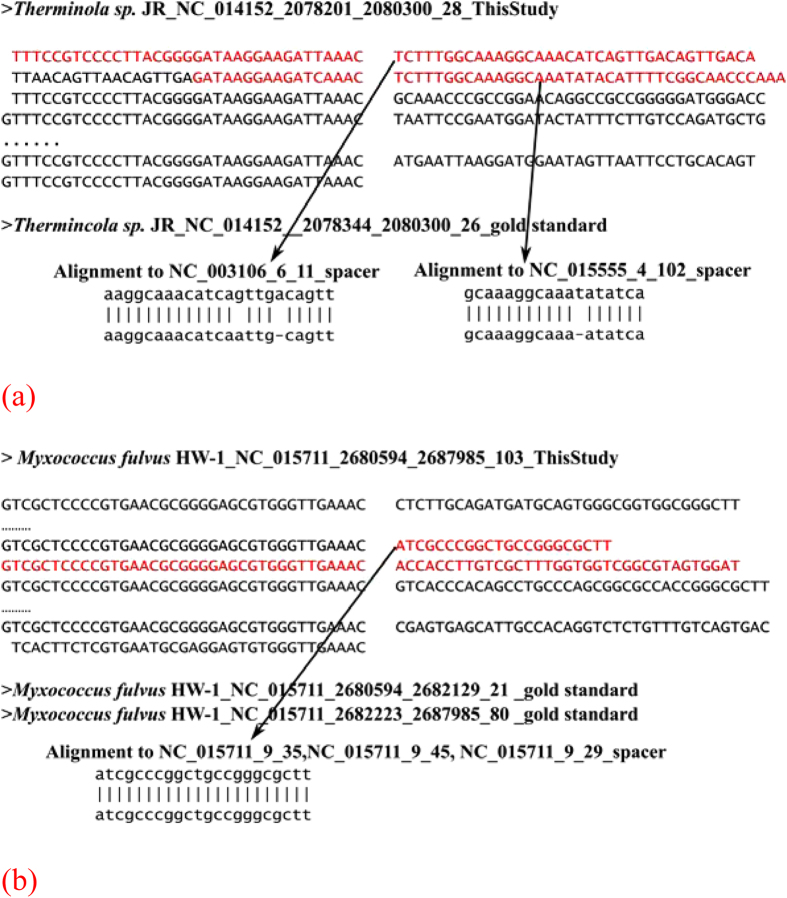
Two examples of novel spacers detected by CRISPRdigger. The two CRISPRs are in the genomes of (**a**) *Therminola sp.* JR, and (**b**) *Myxococcus fulvus* HW-1.

CRISPRdigger detected multiple novel CRISPR spacers, and some of them are supported by their similarities to the known spacer sequences, as shown in Fig. 8. There is a CRISPR in the chromosome region [2078344, 2080300] of *Therminola sp.* JR. CRISPRdigger found a non-standard DR copy with partial match to the other DRs, and expanded this CRISPR with two more candidate spacers, as shown in Fig. 8(a). The two novel spacers were homologous to the known spacers from archaea *Sulfolobus tokodaii* str. 7 DNA and bacteria *Thermoanaerobacterium xylanolyticum* LX-11, respectively. Although both *Therminola sp.* JR and *Thermoanaerobacterium xylanolyticum* LX-11 belong to the taxonomical class Clostridia, there are no homologous spacer copies in the other Clostridia genomes. So it’s probably that an invasive foreign element targeted by the second novel spacer independently invaded these three species, and was captured by the CRISPR machinery. The first novel spacer may have a similar evolutionary history, since *Therminola sp.* JR and *Sulfolobus tokodaii* str. 7 DNA are from different kingdoms.

CRISPRdigger detected an additional DR copy between two CRISPRs in the Deltaproteobacteria *Myxococcus fulvus* HW-1, and these two CRISPRs may be combined into one CRISPR with 103 spacers, as shown in Fig. 8(b). Two novel spacers were detected, and one of them is identical to a spacer in three other CRISPRs in the same genome. The data suggests that the invasive foreign element targeted by this spacer was captured by four CRISPRs independently in *Myxococcus fulvus* HW-1.

### Program running time

CRISPRdigger runs slower than the other three programs, PILER-CR, CRT and CRISPRFinder. Table 1 shows that PILER-CR and CRT run for similar lengths of time on all the four investigated microbial genomes. CRISPRFinder runs much slower than the two programs. CRISPRdigger runs about 50 times slower than CRISPRFinder. The column “RM” in Table 1 explains why CRISPRdigger runs slowly. RepeatMasker is a very good and almost standard repeat screening program, but its running time is slow due to its iterative repeat screening steps. Since the majority of the microbial genomes are shorter than 10 MB, the running speed of CRISPRdigger is acceptable. RepeatMasker-based repeat template mapping step will be a major focus for future improvements, for considering both sensitivity and running speed.

## Conclusion

This work improves CRISPR annotation capacity by providing more stable DR detections, and the procedure is implemented as an easy-to-use CRISPRdigger computer program. CRISPRdigger recovers many more gold standard DRs than the existing programs, but it may miss some very short CRISPRs with only two or three DRs. These short CRISPRs seem to be a challenge for all the four programs. CRISPRdigger does not consider incomplete repeats at the end of a query nucleotide sequence, because it’s difficult to rule out one of the two alternative hypotheses, *i.e.* they belong to two parts of one CRISPR broken by the un-assembled region, or they are two CRISPRs. Rho M. *et al*. proposed a computer program to estimate whether two partial CRISPRs on the sequence boundaries may belong to the same CRISPRs^39^, and long sequencing reads by the third generation sequencing technologies may also facilitate the accurate annotations of long CRISPRs.

So CRISPRdigger provides CRISPR annotations complementary to the existing programs, and the integration of all the programs using optimized parameters may give a more comprehensive landscape of CRISPRs in a given genome.

## Additional Information

**How to cite this article**: Ge, R. *et al*. CRISPRdigger: detecting CRISPRs with better direct repeat annotations. *Sci. Rep.* **6**, 32942; doi: 10.1038/srep32942 (2016).

## Supplementary Material

Supplementary Information

## Figures and Tables

**Table 1:** The running time of four CRISPR detection programs on four microbial genomes.

Genome	Gsize (MB)	PILER-CR	CRT	CRISPRFinder	CRISPRdigger		
					RM	All	All-RM
Neisseria_NC_017512	2.207	<1	<1	7	137	144	7
Sulfolobus_NC_013769	2.697	1	<1	3	163	169	6
Salmonella_NC_017623	4.706	2	<1	4	218	220	2
Salmonella_NC_021820	4.777	1	<2	4	221	223	2

Column “Gsize” gives the genome size in megabase pairs (MB). The columns “PILER-CR”, “CRT”, “CRISPRFinder” and “CRISPRdigger” give the running time of the programs in seconds. The RepeatMasker utilized in CRISPRdigger consumes a large proportion of running time, and its running time is estimated separated in the column “RM”. The column “All” gives the total running time of CRISPRdigger, and the column “All-RM” gives the difference between the two columns “RM” and “All”.
