# Supplementary material for: CRISPRdigger: detecting CRISPRs with better direct repeat annotations
Source: Sci Rep. 2016 Sep 6;6:32942. doi: 10.1038/srep32942 (PMC5011713; doi:10.1038/srep32942)
Supplement: Supplementary Information [file srep32942-s1.pdf]

CRISPRdigger: detecting CRISPRs with better direct repeat annotations

Ruiquan Ge[1,2,*], Guoqin Mai[1,*], Pu Wang[1,2], Manli Zhou[1,2], Youxi Luo[3], Yunpeng Cai[1,#], Fengfeng Zhou[4,5,#].

[1] Shenzhen Institutes of Advanced Technology, and Key Lab for Health Informatics, Chinese Academy of Sciences, Shenzhen, Guangdong 518055, China.

[2] Shenzhen College of Advanced Technology, University of Chinese Academy of Sciences, Shenzhen, Guangdong, 518055, China.

[3] School of Science, Hubei University of Technology, Wuhan, Hubei, 430068, China.

[4] College of Computer Science and Technology, Changchun, Jilin, 130012, China.

[5] Key Laboratory of Symbolic Computation and Knowledge Engineering of Ministry of Education, Jilin University, Changchun, Jilin 130012, China.

[*] These authors contribute equally to this work.

[#] Corresponding author: Fengfeng Zhou, e-mail: FengfengZhou@gmail.com or ffzhou@jlu.edu.cn .Web site: http://www.healthinformaticslab.org/ffzhou/.

Supplementary Figure S1

The work flow of the program CRISPRdigger. Below is the algorithmic flow of the program CRISPRdigger. Input files should be in the FASTA format. Direct Repeats (DRs) were mainly found by RepeatScout and RepeatMasker. According to the character and the restriction of the DRs and spacers, parts of the CRISPRs were identified. After extending the DRs, the CRISPRs were enlarged or combined. Result files included the detailed information of the CRISPRs.

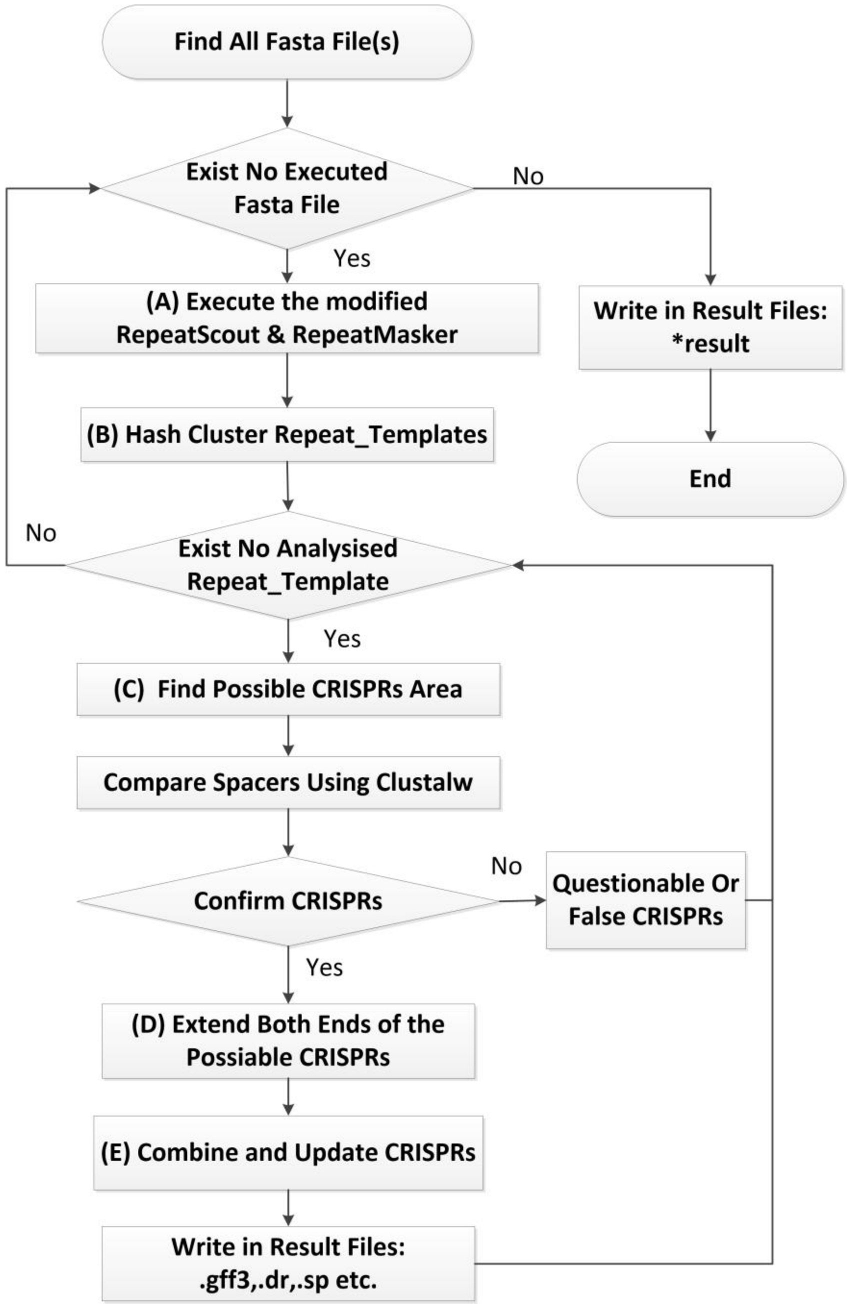

Supplementary Figure S2

The comparison on the top 10 prokaryotic genus with the largest CRISPR numbers. The top 10 prokaryotic genus with the largest CRISPR numbers are used to analyze in dbCRISPR. The bottom figures show their CRISPRs statistical distributions in the four CRISPR detection programs: CRISPRdigger, CRT, PILER-CR and CRISPRFinder.

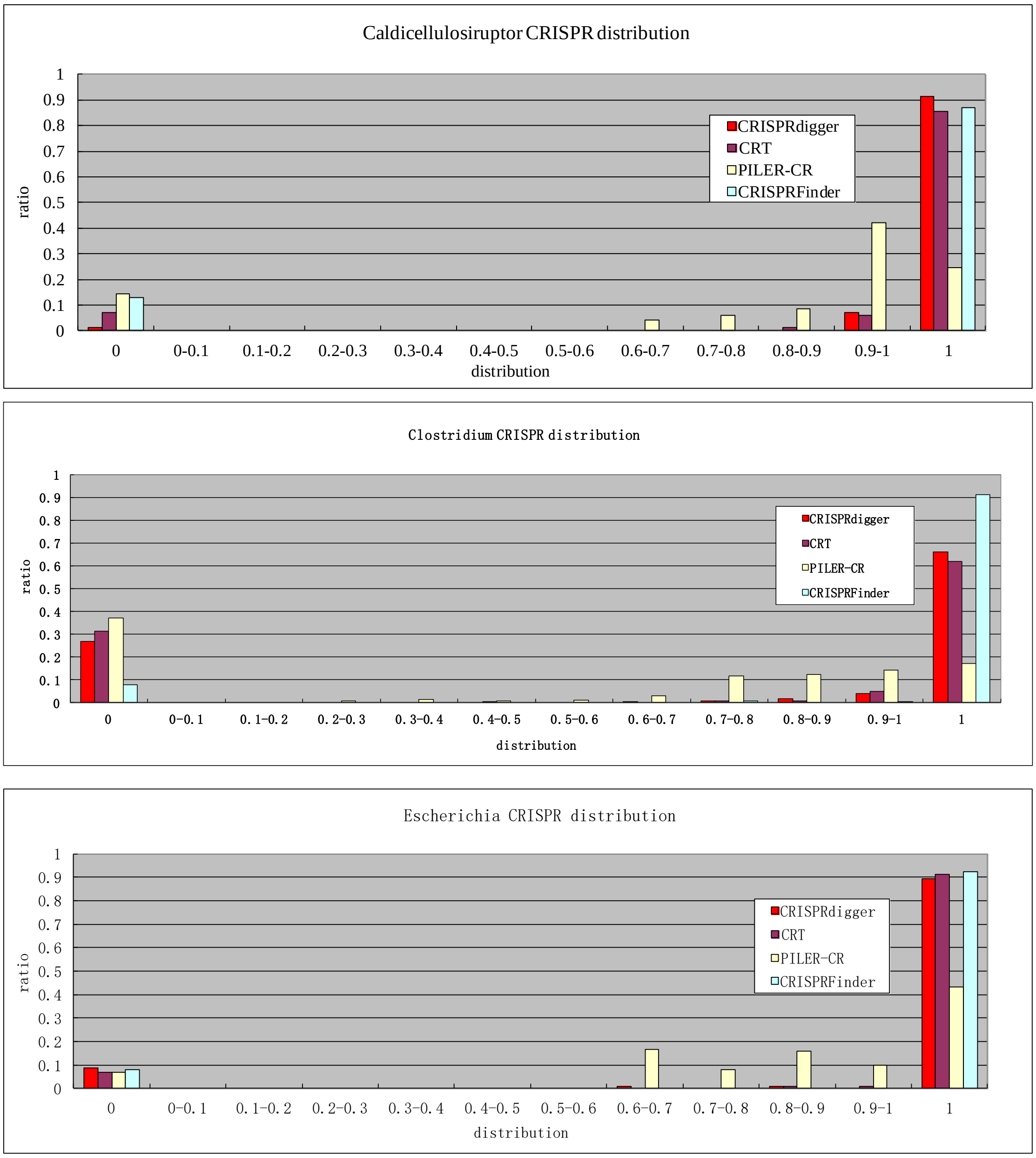

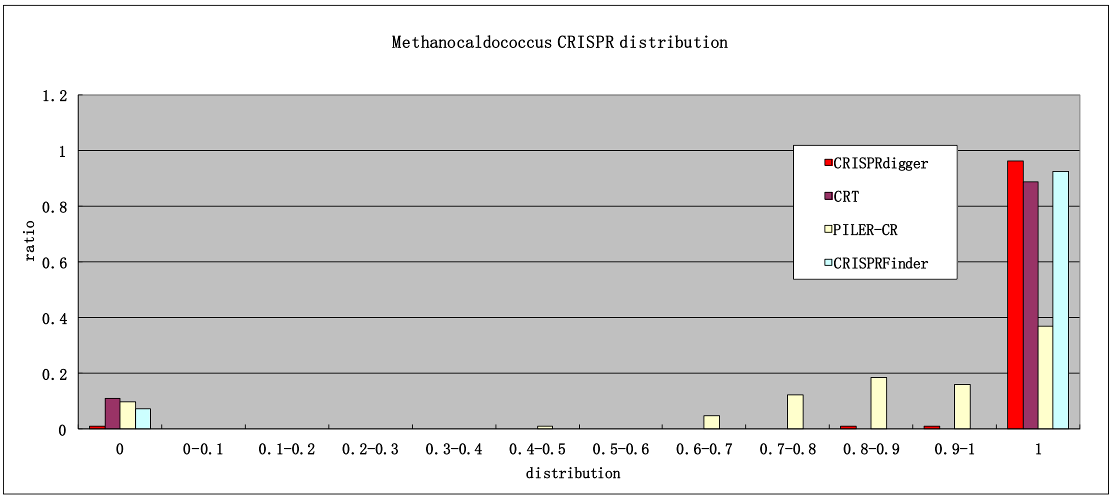
Methanocaldococcus CRISPR distribution
ratio
1.2
1
0.8
0.6
0.4
0.2
0
CRISPRdigger
CRT
PILER-CR
CRISPRFinder
0
0-0.1
0.1-0.2
0.2-0.3
0.3-0.4
0.4-0.5
0.5-0.6
0.6-0.7
0.7-0.8
0.8-0.9
0.9-1
1
distribution

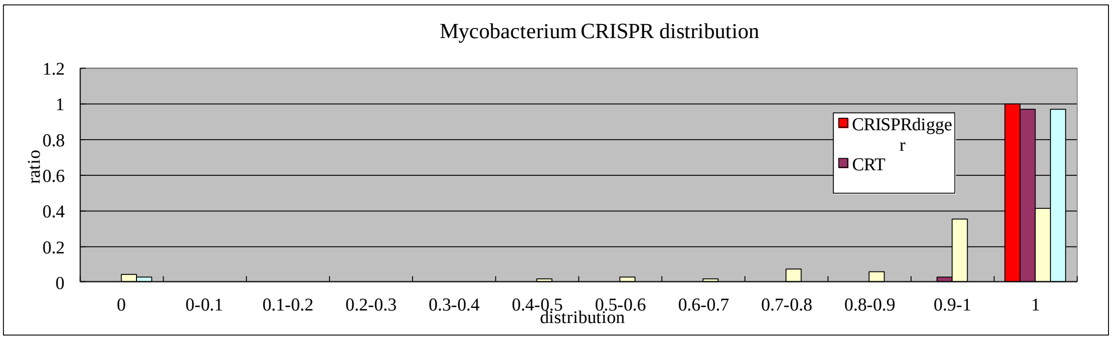
Mycobacterium CRISPR distribution
ratio
1.2
1
0.8
0.6
0.4
0.2
0
CRISPRdigge r
CRT
0
0-0.1
0.1-0.2
0.2-0.3
0.3-0.4
0.4-0.5
0.5-0.6
0.6-0.7
0.7-0.8
0.8-0.9
0.9-1
1
distribution

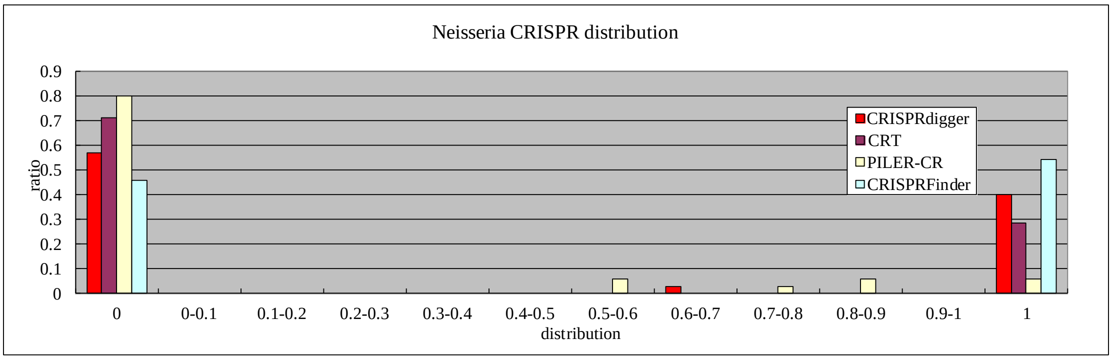
Neisseria CRISPR distribution
ratio
0.9
0.8
0.7
0.6
0.5
0.4
0.3
0.2
0.1
0
CRISPRdigger
CRT
PILER-CR
CRISPRFinder
0
0-0.1
0.1-0.2
0.2-0.3
0.3-0.4
0.4-0.5
0.5-0.6
0.6-0.7
0.7-0.8
0.8-0.9
0.9-1
1
distribution

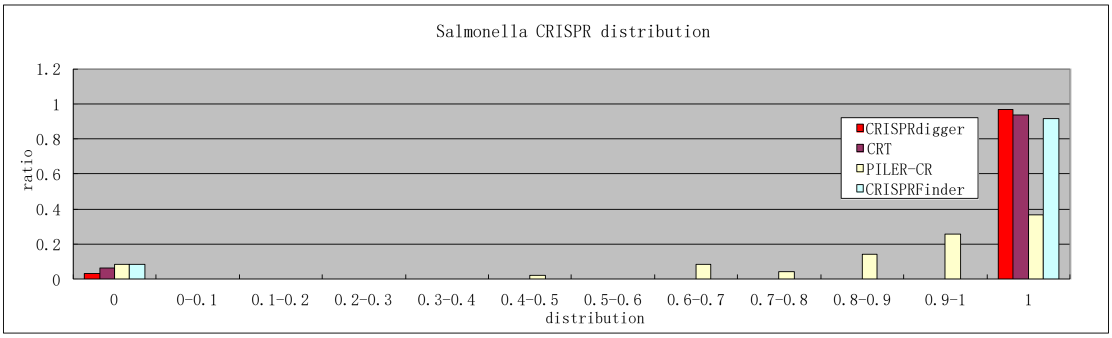
Salmonella CRISPR distribution
ratio
1.2
1
0.8
0.6
0.4
0.2
0
CRISPRdigger
CRT
PILER-CR
CRISPRFinder
0
0-0.1
0.1-0.2
0.2-0.3
0.3-0.4
0.4-0.5
0.5-0.6
0.6-0.7
0.7-0.8
0.8-0.9
0.9-1
1
distribution

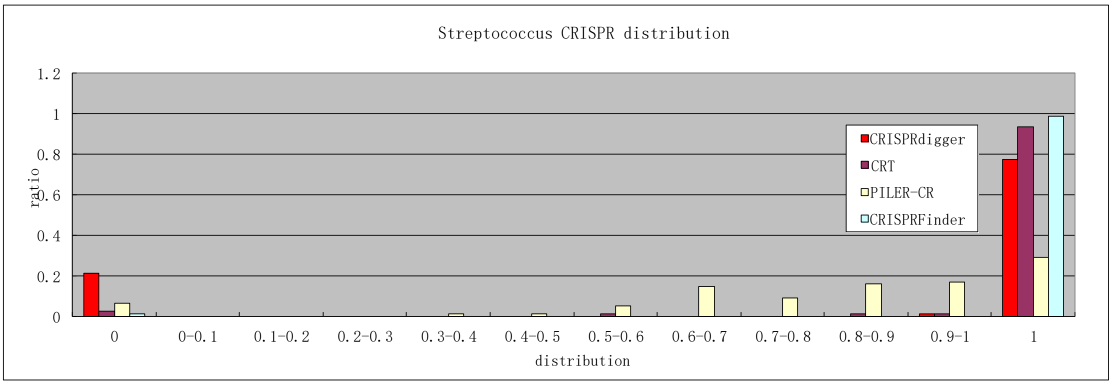
Streptococcus CRISPR distribution
1.2
1
0.8
0.6
0.4
0.2
0
ratio
0
0-0.1
0.1-0.2
0.2-0.3
0.3-0.4
0.4-0.5
0.5-0.6
0.6-0.7
0.7-0.8
0.8-0.9
0.9-1
1
distribution
CRISPRdigger
CRT
PILER-CR
CRISPRFinder

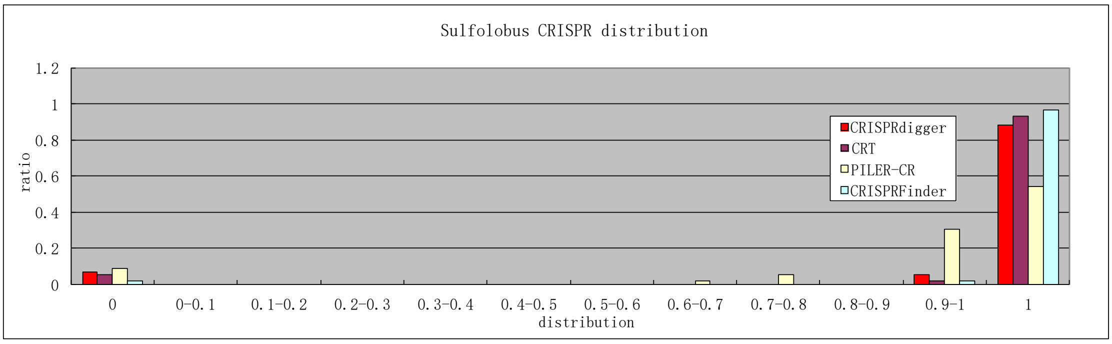
Sulfolobus CRISPR distribution
1.2
1
0.8
0.6
0.4
0.2
0
ratio
0
0-0.1
0.1-0.2
0.2-0.3
0.3-0.4
0.4-0.5
0.5-0.6
0.6-0.7
0.7-0.8
0.8-0.9
0.9-1
1
distribution
CRISPRdigger
CRT
PILER-CR
CRISPRFinder

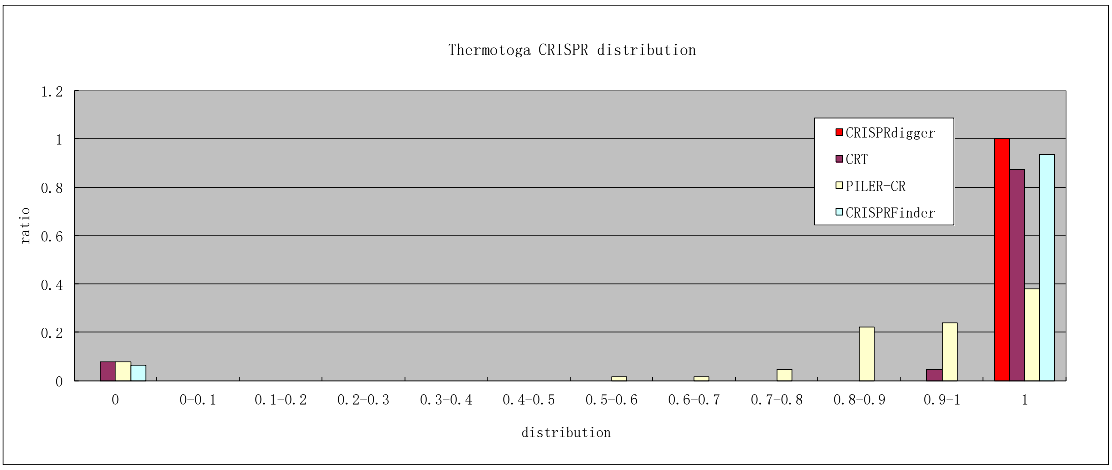
Thermotoga CRISPR distribution
1.2
1
0.8
0.6
0.4
0.2
0
ratio
0
0-0.1
0.1-0.2
0.2-0.3
0.3-0.4
0.4-0.5
0.5-0.6
0.6-0.7
0.7-0.8
0.8-0.9
0.9-1
1
distribution
CRISPRdigger
CRT
PILER-CR
CRISPRFinder
